# Influence of mouth rinses on the surface hardness of dental resin nano-composite

**DOI:** 10.12669/pjms.316.7611

**Published:** 2015

**Authors:** Aftab Ahmed Khan, Adel Zia Siddiqui, Syed Fareed Mohsin, Abdulaziz A. Al-Kheraif

**Affiliations:** 1 Aftab Ahmed Khan, MSc, M.Bioeth, B.D.S. Researcher, Dental Biomaterials Research Chair, College of Applied Medical Sciences, King Saud University, Riyadh 11433; Saudi Arabia; 2 Adel Zia Siddiqui, MSc, B.D.S. Associate Professor, Department of Dental Material Sciences, Baqai Dental College, Baqai Medical University, 51 Deh Tor, Toll Plaza, Super Highway, Gadap Road, Karachi 74600; Pakistan; 3 Syed Fareed Mohsin, Ph.D, MSc, MFD RCS, MFDS RCPSG, B.D.S. Associate Professor, Department of Oral Pathology/Oral Medicine, Baqai Dental College, Baqai Medical University, 51 Deh Tor, Toll Plaza, Super Highway, Gadap Road, Karachi 74600; Pakistan; 4 Abdulaziz A. Al-Kheraif, Ph.D, MSc. Associate Professor & Dental Biomaterials Research Chair, College of Applied Medical Sciences, King Saud University, Riyadh 11433; Saudi Arabia

**Keywords:** Hardness, Microscopy, Mouth rinses, Nano-filled composite

## Abstract

**Objective:**

The aim of this research was to assess the effect of mouth rinses with and without alcohol on the hardness of dental nano-filled composite.

**Methods:**

The micro-hardness of fifty circular disk shaped specimens of 7 mm x 2 mm were measured after 14 days. Specimens were immersed into alcohol containing (Listerine and Colgate Perioguard) and alcohol-free (Prodent and Sensodyne Oral antiseptic) mouth rinse solutions. Artificial saliva served as the control. Vickers Micro-hardness was measured with a 30gram load for 30 seconds dwell time by using a diamond indenter. Significant differences were represented by p<0.05, whereas highly significant difference represented by p<0.01. The level of significance (p) was calculated with the help of repeated measure ANOVA. For multiple comparisons, Tukey’s multiple comparison test was used.

**Results:**

Statistical analysis revealed highly significant difference between specimens immersed in artificial saliva (control) and Listerine (p<0.01). Whereas significant difference were observed between control and Colgate Periogard (p<0.05). However, no significant difference was observed on comparing Prodent and Sensodyne Oral antiseptic mouth rinses with control group(p>0.05). Control specimens depicted highest value of micro-hardness(60.5746 ± 3.2703) compared to the lowest value seen in specimens immersed in Listerine solvent(54.4687 ± 1.0937).

**Conclusion:**

Alcohol containing mouth rinsing solutions have more deleterious effect on hardness of nano composites as compared to alcohol-free mouth rinses.

## INTRODUCTION

Dental composite is a material of choice for direct restorations.^1^,^2^ The chemical action of organic solvents associated with lack of good mechanical and physical properties which makes it liable to suffer from dissolution and degradation in the oral cavity. This leads to surface roughness and decreased hardness of the material.^1^

Although the initial hardness of the polymerized composite material is sufficient to withstand the masticatory load yet the degradation process that initiates immediately in the oral cavity make the composite material vulnerable to dissolution followed by disintegration. The deterioration of a composite material is an intricate process dependent on the filler quantity, matrix type and the coupling agent used in the material. The action of food and organic solvents, water uptake by the matrix^3^, thermal changes, mechanical cycling and the characteristics of the oral cavity are some of the factors that influence surface degradation, crack propagation and hardness of the material.^4^ The surface integrity of composite material is directly related to clinical longevity of the composite material.^5^,^6^

In recent years, the use of mouth rinses has increased tremendously to thwart action against plaque, caries and periodontal diseases.^7^ It is an effective method for oral hygiene maintenance.^8^ These mouth rinses contain water, antimicrobial agents, detergents, emulsifiers and organic acids and in some cases alcohol.^9^ Changing the concentration of these substances alter the oral pH.^10^ Studies have shown influence of alcohol containing mouth rinses on the surface roughness and hardness of the composites.^8^,^10^,^11^ On the contrary there are studies claiming to have no adverse effect of alcohol mouth rinses on the hardness of the composite material^12^ and assert that micro hardness value depends on the material itself rather than the rinsing solutions used.^11^

Surface hardness is an important physical property which correlates well with the mechanical properties such as abrasion resistance and compressive strength of the material.^13^ It is assumed that clinical longevity and aesthetic of the restoration is very much dependent on this property.^13^ Therefore our objective of the study was to investigate the effect of commonly available mouth rinses on the surface hardness of the nano-filled composite material.

## METHODS

The composite resin used for preparing 50 circular disk shaped specimens of 7 mm x 2 mm thickness each was Te- Econom Plus (IvoclarVivadent, UK). Teflon mold was used in fabricating the specimens. Mylar strip (Dentart, Polidental, Sao Paulo, Brazil) having dimensions of 10 × 120 × 0.05 mm was placed over the top and bottom of the mold and pressed from top with a microscope slide 22 × 22 mm (BDH borosilicate glass) to form a flat surface of the specimen. Light curing unit Quartz Tungsten Halogen (401™ Demetron Research Corporation, Danbury, CT, USA) with light intensity of 550 W/cm^2^ was held rigidly and placed 1.0 mm over the glass slide for 40 seconds to polymerize the specimens. Sof-Lex (3M ESPE, USA)and Sof-Lex discs were used to polish specimens to get a clinical finish. These fabricated specimens were placed in distilled water for 24 hours for post irradiation hardness. After 24 hours specimens were dried with absorbing paper and ready for immersion into the control and experimental solutions.

Specimens were randomly divided into five groups. Each group containing 10 specimens (n=10). The active ingredients of the control are displayed in Table-I and the experimental groups are displayed in Table-II with their respective pH. The specimens were immersed in 10 ml of respective solutions. The specimens of group 1 (control) were stored in artificial salivaat 37±3°C in an incubator (Sanfa DNP-9052, China) for 14 days. Likewise, specimens of group 2 were stored in Listerine (alcohol containing); group 3 in Colgate Periogard (alcohol containing); group 4in Prodent (alcohol free); and group 5 in Sensodyne Oral Antiseptic (alcohol free)respectively.

**Table-I:** Showing different ingredients of Artificial Saliva along with pH

S. No.	Solvent	Ingredients	pH
1	Artificial Saliva	Sodium chloride (NaCl) 0.400 g; Potassium chloride (KCl) 0.400 g; Calcium chloride monohydrate (CaCl_2_H_2_O) 0.795g; Sodium dihydrogen phosphate (NaH_2_PO_4_) 0.69 g; Disodium sulphide hydrate (Na_2_Sx9H_2_0) 0.005 g; Urea 1.0 g	6.9

**Table-II:** Showing the active components of different mouth rinsing solutions with their pH andalcoholic content.

Mouth rinse	Manufacturer	Components	Indications/Treatment	pH	Alcohol Content
Listerine	Johnson & Johnson Instrustiral Ltd., Soa Paulo, Brazil	Eucalyptol 0.092%, Menthol 0.042%, Methyl Salicylate 0.060, Thymol 0.064%, Water, Sorbitol, Alcohol, Poloxamer 407, Flavor, Benzoic acid, Stem extract, Sodium Benzoate	Antiplaque, Mild Anti gingivitis effect.	4.3	21.6%
Colgate Periogard	Colgate Palmolive Industria e ComercioLtda, Soa Paulo, Brazil	0.12% Chlorhexidinegluconate,Water, Alcohol, Glycerin, PEG-40 sorbitandiisostearate, flavor, Sodium saccharin.	Antimicrobial, Anti fungal, Broad spectrum antiseptic, Bactericidal against Gram +ve and Gram –ve, Denture stomatitis.	5.6	11.6%
Prodent	Platinum PhamaceuticalsPvt Ltd, Pakistan	Potassium nitrate 1%	Dentinal hypersentivity, Anti inflammatory and bleeding gums.	6.2	Alcohol free
Sensodyne Oral antiseptic	Glaxo Smith Kline, Rio de Janeiro, Brasil	Cetylpyridinium chloride 0.05%, Sodium fluoride 226 ppm F, Water, Glycerine, Sorbitol 70%, Poloxamer 338, PEG-60, Hydrogenated castor oil, Sodium benzoate, Flavouring, Methylparaben, Sodium saccharin, Sodium Phosphate, Disodium Phosphate	Antiplaque, Sensitive teeth, Halitosis.	6.1	Alcohol free

### Scanning Electron Microscopy (SEM) Analysis

Surface changes were observed using the scanning electron microscopy (SEM) at 20 kV accelerating voltage. The specimens were mounted on aluminum stubs, sputter-coated with gold and examined with a scanning electronic microscope (JEOL-JSM; 6460LV, Tokyo, Japan).

### Vickers Micro-hardness Test

Micro-hardness was measured for all the 50 specimens by using a 50gram load for 30 seconds dwell time. A square base pyramid shaped diamond micro indenter of136° was used. Each specimen was indented 3 times at different places and a mean value was obtained for each disk. Micro-hardness values of the specimens were recorded using Vickers micro hardness tester (MMT – X7 Matsuzuwa, Japan).

### Statistical Analysis

Data was entered in Statistical Package for Social Sciences (SPSS) version 19. Descriptive analysis was executed in the form of mean ± standard deviation for surface micro-hardness. p<0.05 were considered to be significant. Significant differences were represented by p<0.05, whereas highly significant difference represented by p<0.01. The level of significance (p) was calculated with the help of repeated measure ANOVA. For multiple comparisons, Tukey’s multiple comparison test was used.

## RESULTS

Statistical analysis of the data revealed highly significant difference (p<0.01) between the control and Listerine group while a significant difference was observed when control was compared to Colgate Periogard. However, no significant differences were observed on comparing Prodent and Sensodyne Oral antiseptic with control. Control specimens demonstrated highest value of micro-hardness, however, on the other hand specimens immersed in the Listerine solution were found to have the lowest micro-hardness values. Furthermore, Colgate Periogard demonstrated a reduction in micro-hardness which was also significant from control. Whereas, no significant change was observed for specimens in Prodent or in Sensodyne Oral Antiseptic solutions. Actual p values of treatment versus control and mean values of micro hardness test with their respective standard deviations are presented in Table-III.

**Table-III:** Showing the mean and standard deviation values of Vickers micro-hardness testing.

Groups	Immersing Medium	Mean ± SD	p-value
1	Artificial Saliva	60.5746 ± 3.2703	Control
2	Listerine	54.4687 ± 1.0937	2.58683E-05
3	Colgate Periogard	58.0366 ±0.53798	0.026236797
4	Prodent	60.0057 ± 0.81027	0.599905294
5	Sensodyne Oral antiseptic	59.8782 ± 0.90889	0.524650214

**Fig.1:**
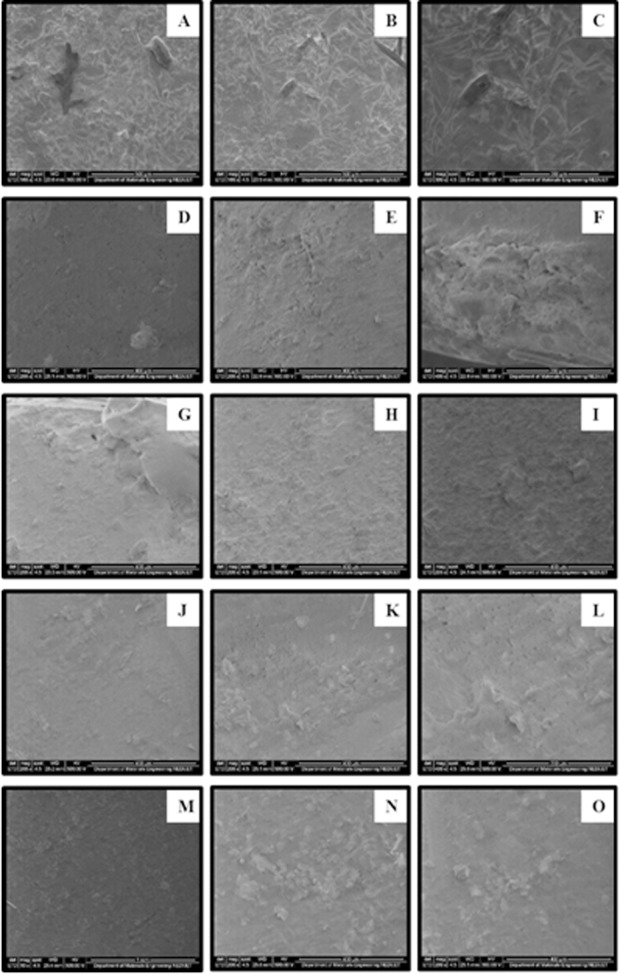
SEM micrographs of nano-filled composite specimens (A-C) specimen immersed in Artificial Saliva at 100 x, 200 x and 300 x respectively, (D-F) specimen immersed in Listerine at 100 x, 200 x and 300 x respectively, (G-I) specimen immersed in Colgate Periogard at 100 x, 200 x and 300 x respectively, (J-L) specimen immersed in Prodent at 100 x, 200 x and 300 x respectively, (M-O) specimen immersed in Sensodyne Oral Antiseptic at 100 x, 200 x and 300 x respectively.

The SEM micrographs examination revealed presence of micro cracks and voids in the specimens of control and experimental groups (Fig.1). A surface alteration can be observed in micrographs (A-C) of control group. The most evident porosity and cracks were observed in the micrographs (D-F) of Listerine group. The results of this study proved that alcohol containing mouth rinses have significant effect on the hardness values.

## DISCUSSION

The ideal restorative material is the one that functions in the same manner as our natural teeth do under different masticatory loads with changing oral environment. Besides, these materials should closely resemble in appearance with that of the natural teeth.^14^ The quest for new material with these ideal properties has already begun. Nano-filled composites are the newest addition to the pantheon of composite filling materials; emerged as suitable alternatives to overcome limitations that other types of composites have.^15^ Nano-filled composites are considered to be the material of choice because of their improved physical and mechanical properties are not found in other types of composites.^15^ This study involved one of these type of new composite material (Te Econom Plus) to evaluate the micro hardness which is considered an important property for the longevity of the composite material.

To evaluate the effect of alcoholic mouth rinses on the micro hardness of nano composite, this study involved two different commercially available mouth rinses; and their effect on nano composites were compared with alcohol free mouth rinses. The results of this study found that alcohol concentration has a direct influence on one of the physical property ofthe material i.e. hardness (Table-III). The reason could be the low pH of organic solvents like alcohol which have the tendency to damage polymer chain. Alcohol can penetrate into the polymeric chain and thereby causing the release of unreacted monomers.^16^,^17^ Alcohol has the affinity to penetrate the polymer chain and damage it in no time. The low pH of alcohol mouth rinses catalysis the ester groups from dimethacrylate monomers present in the composite which leads to destruction of the polymer chain.^17^ This is followed by hydrolytic degradation of the composite material.^12^

Continual storage of specimens for 14 days in different mouth rinsing solutions significantly decreased the micro hardness of the composite material in every group. This surface change can be observed with SEM micrographs (Fig 1). Matrix cracking and its propagation in a resin material is attributed to water uptake process. This vicious circle of a composite material starts initially with the swelling of a composite material and leads to interfacial debonding and dislodgment of filler particles over the period of time.^18^ This hydrolytic degradation mechanism aggravates if fillers have metallic ions present in them.^19^ The fillers such as barium and zinc are electropositive in nature and have affinity to react with water. Loss of these elements into water perturbs the charge balance of silica network. Silica network reestablishes the charge balance with the penetration of hydrogen ions. This process results into breakage of (Si-O-Si) bonds leading to softening of the composite resin with aging time.^19^ The results of this study justified this hydrolytic degradation mechanism. The presence of barium fillers in the nano-filled composite we used further testified the results and degradation process in all the groups.

The results of our study are consistent with the previous studies of Miranda *et al*.^10^ who found decreased values of micro-hardness due to alcohol presence; Almeida *et al*.^8^ who observed severe degradation of nano-filled composite due to mouth rinses; and Jyothi *et al*.^20^ work which found significant reduction in mean VHN (Vickers micro hardness number) of specimens immersed in alcohol based mouth rinses. SEM micrographs further validate this effect on the hardness of the nano-filled composites.

Viewing the results of present data confirmed that alcohol containing mouthrinses showed major influence on micro-hardness values when compared with control. The limitation of our study was to create a dynamic oral environment that cannot be exactly and entirely replicated by in vitro laboratory conditions.^21^ Further studies need to be conducted to access longer exposure times using artificial saliva and other solvents in order to mimic the clinical oral condition. Also other properties such as tensile testing and colour stability may be conducted to provide more specific data about the effects of pH and alcohol concentration of various solvents on esthetic restorative materials.^22^

## CONCLUSION

We can conclude from this *in vitro* study that:

- The surface hardness values of the nano composites are differently affected by the mouth rinse solutions
- Degradation was observed in all the specimens during this study.
- Alcohol containing mouth rinses showed more reduction in micro hardness values of nano-composite as compared to alcohol free mouth rinses.
